# Obesity needs to be addressed to tackle the increased prevalence of diabetes in China – Temporal changes from 2003 to 2009

**DOI:** 10.1016/j.pmedr.2021.101625

**Published:** 2021-10-25

**Authors:** Shahina Pardhan, Dingchang Zheng, Zhiqing Chen, Guillermo F. López Sánchez

**Affiliations:** a Vision and Eye Research Institute, School of Medicine, Faculty of Health, Education, Medicine and Social Care, Anglia Ruskin University, Cambridge CB1 1PT, United Kingdom; b Faculty of Health and Life Sciences, University of Coventry, Coventry, United Kingdom; c Eye Centre, the Second Affiliated Hospital of the School of Medicine, Zhejiang University, 88 Jiefang Road, Hangzhou, Zhejiang 310009, PR China; d Key Laboratory of Ophthalmology of Zhejiang Province, 88 Jiefang Road, Hangzhou, Zhejiang 310009, PR China

**Keywords:** Diabetes, Prevalence, Risk factors, Protective factors, China

## Abstract

This study aimed to analyse the temporal change of diabetes and any associated risk and protective factors for diabetes in Chinese adults between Wave 0 (2003) and Wave 1 (2009) of the World Health Organization (WHO) Study on global AGEing and adult health (SAGE).

Data from China of the SAGE were analysed. Diabetes (outcome variable) was assessed by the yes/no question: “Have you ever been diagnosed with diabetes (high blood sugar)?”. Exposure variables examined in bivariate and multivariate multiple regression included sex, age, marital status, education, smoking, alcohol, fruit and vegetables consumption, physical activity and body mass index (BMI). Significant exposure variables in bivariate analyses were included in multivariate analyses (2003: age and tobacco; 2009: age, BMI, education and alcohol).

In Wave 0 (2003), there were 3993 Chinese adults, of which 67 (1.7%) self-reported to have diabetes. In Wave 1 (2009), there were a total of 9524 Chinese adults, of which 770 (8.1%) had diabetes. The overall prevalence of diabetes in Chinese adults increased by 4.76 times between the two timeframes (1.7%, age range 27–84 years, average age 58.51 ± 12.70 years, 59.70% females in 2003 to 8.1%, age range 20–95 years, average age 65.31 ± 10.19 years, 53.64% females in 2009). Multivariate regression retained older age ≥ 60 years (OR 4.34, 95% CI 2.67–7.07) as the main risk factor in 2003 data, while in 2009 the odds ratio for older age ≥ 60 years decreased (OR 2.45, 95% CI 2.06–2.92), but included a significant association of obesity (OR 2.11, 95% CI 1.60–2.78) and excess weight (OR 1.42, 95% CI 1.19–1.69).

The significant association with excess weight and obesity associated with the increased prevalence of diabetes in 2009 is a cause of concern and should be addressed by public health strategies in China.

## Highlights

• The overall prevalence of diabetes in Chinese adults increased by 4.76 times between the two timeframes (1.7% in 2003 to 8.1% in 2009).
• In 2003, older age ≥ 60 years (OR 4.34, 95% CI 2.67–7.07) was the main risk factor.
• In 2009, the odds ratio for older age ≥ 60 years decreased (OR 2.45, 95% CI 2.06–2.92), but included a significant association of obesity (OR 2.11, 95% CI 1.60–2.78) and excess weight (OR 1.42, 95% CI 1.19–1.69).
• The significant association with excess weight and obesity associated with the increased prevalence of diabetes in 2009 is a cause of concern and should be addressed by public health strategies in China.

## 1 Introduction

Approximately 422 million people worldwide had diabetes in 2020 (World Health Organization, 2020a). According to cross sectional data from 2015 to 2017, China has the highest number of people with diabetes mellitus in the world, with an estimated number of patients with diabetes in mainland China of 129.8 million (70.4 million men and 59.4 million women) (Li et al., 2020). This suggests that 30% of the people with diabetes of the world are from China. Other nationally representative studies suggest that the prevalence of diabetes is increasing progressively in China: 9.7% in 2007/2008 (Yang et al., 2010), 10.9% in 2013 (Wang et al., 2017) and 12.8% in 2015/2017 (Li et al., 2020).

As diabetes has become a major public health problem in the general population of China (Xu et al., 2013), prevention and good management of diabetes is of paramount importance in that country. To be able to do this, it is necessary to understand the specific risk (and protective) factors that may be associated with the increased prevalence of diabetes in China. As diabetes is multifactorial (Hansen, 2002) it would be important to explore whether the risk factors also change with any temporal change in prevalence. It is known that several factors can directly affect the risk of developing diabetes, such as age, body mass index, diet, physical activity, smoking or alcohol, although there are some discrepancies among studies and more research is still needed in this field (Centers for Disease Control and Prevention, 2021).

Furthermore, although some previous studies have analysed the prevalence of diabetes and its risk factors in China (Li et al., 2020, Wang et al., 2017, Yang et al., 2010), there is a paucity of studies exploring the evolution of the significant risk factors associated with the temporal changes of diabetes in China. Such information is necessary to aid in the development of targeted diabetes prevention strategies in China, especially considering that the economic burden of diabetes has recently increased in China (Huang et al., 2016). The aim of this study was to analyze the temporal changes of the prevalence and associated risk and protective factors of diabetes in Chinese adults, from nationally representative studies, between 2003 (WHO’s World Health Survey 2003- also called WHO’s Global Ageing and Adult Health Wave 0) and 2009 (WHO’s Global Ageing and Adult Health- termed Wave 1). We hypothesize that there would be an increase in the prevalence of diabetes from 2003 to 2009, as this has been shown around the world, as people are living longer and also as people are leading more sedentary lifestyles as a result of economic progress.

## 2 Methods

### 2.1 The survey

This study used data of Waves 0 and 1 of the World Health Organization (WHO) Study on global AGEing and adult health (SAGE) (http://www.who.int/healthinfo/sage/en/). Details of the SAGE survey methodology have been published previously (Kowal et al., 2012). In brief, a multistage clustered sampling design method was used to obtain nationally representative samples. The sample consisted of adults aged ≥ 18 years with oversampling of those aged ≥ 50 years. Trained interviewers conducted face-to-face interviews using a standard questionnaire. Sampling weights were constructed to adjust for the population structure as reported by the United Nations Statistical Division. Ethical approval for data collection was obtained from the WHO Ethical Review Committee and local ethics research review boards and written informed consent was obtained from all participants (Kowal et al., 2012).

### 2.2 Participants

In Wave 0, there were 3993 Chinese adults (age range 18–99 years, average age 45.12 ± 15.53 years, 51.06% females), of which 67 self-reported to have diabetes (age range 27–84 years, average age 58.51 ± 12.70 years, 59.70% females). In Wave 1 (conducted only six years later), there were a total of 9524 Chinese adults (age range 18–104 years, average age 60.89 ± 12.39 years, 53.80% females), of which 770 had diabetes (age range 20–95 years, average age 65.31 ± 10.19 years, 53.64% females).

### 2.3 Outcome variable

Diabetes was assessed with the following yes/no question: “Have you ever been diagnosed with diabetes (high blood sugar)?”. Previous research has confirmed the validity and high accuracy of self-reported diagnosis of diabetes (Pastorino et al., 2015, Huerta et al., 2009, Schneider et al., 2012).

### 2.4 Exposure variables

The selection of the exposure variables was based on bivariate analyses and previously reported significant variables for diabetes research including: sex, age, BMI, marital status, education, tobacco, alcohol, fruit and vegetables consumption, and levels of physical activity (Laakso and Pyorala, 1985, Yang et al., 2016, American Diabetes Association, 2020, Leong and Wilding, 1999, Scheen, 2000, Duncan et al., 2009, Teratani et al., 2012, Ford and Mokdad, 2001, González-Carcelén et al., 2020, López et al., 2020). Age was categorized according to the WHO definition of older adults (World Health Organization, 2018) as < 60 years and ≥ 60 years. Although Wave 1 had measured weight and height and self-reported weight and height, there were a lot of data missing in the measured weight and height (total sample: n = 1501, 15.8%; sample with diabetes: n = 121, 15.7%) and, therefore, to compare data from the two time frames, self-reported weight and height data were used. BMI was calculated using self-reported weight and height, and it was divided in three categories (World Health Organization, 2021b): <25.0 kg/m^2^ (underweight / normal weight), 25.0–29.9 kg/m^2^ (overweight) and ≥ 30.0 kg/m^2^ (obesity). Current marital status was categorized as never married / separated / divorced / widowed and currently married / cohabiting. Highest level of education completed was divided in three categories: up to primary school, up to secondary school, and college / pre-university / university completed / post graduate degree completed. In wave 0, tobacco use was ascertained by the question Wave 0: Do you currently smoke any tobacco products such as cigarettes, cigars, or pipes? In wave 1, tobacco use was assessed with the question “Do you currently use (smoke, sniff or chew) any tobacco products such as cigarettes, cigars, pipes, chewing tobacco or snuff?”. Responses from both questions were categorized as: daily; yes, but not daily; no, not at all. Alcohol was evaluated with the question “Have you ever consumed a drink that contains alcohol (such as beer, wine, spirits, etc.)?” and the response options were yes and never. Fruit and vegetables consumption was measured through the questions “How many servings of fruit do you eat on a typical day?” and “How many servings of vegetables do you eat on a typical day?”, and participants were categorized as those eating < 5 servings/day and ≥ 5 servings/day (World Health Organization, 2020b, United Kingdom National Health Service, 2018). Physical activity was evaluated using two validated questionnaires: SAGE Wave 0 used the International Physical Activity Questionnaire (IPAQ) Short Form (Craig et al., 2003, Rodriguez-Munoz et al., 2017) and SAGE Wave 1 used the Global Physical Activity Questionnaire (GPAQ) (Bull et al., 2009, Armstrong and Bull, 2006). Although these are different instruments, the unit of physical activity used in both questionnaires is MET-minutes/week, where MET is the Metabolic Equivalent of Task. The IPAQ formula to calculate total physical activity MET-minutes/week was: sum of Walking + Moderate + Vigorous MET-minutes/week scores (IPAQ group, 2005). The GPAQ formula to calculate total physical activity MET-minutes/week was: sum of the total MET minutes of activity computed for each setting (work, travel to and from places, recreational activities) (World Health Organization, 2021a). A study published about concurrent validity between IPAQ and GPAQ showed a moderate to strong positive relationship (Bull et al., 2009). The physical activity cutoff value was 600 MET-minutes/week, dividing the participants in two categories according to current physical activity guidelines (IPAQ group, 2005, World Health Organization, 2021a, World Health Organization, 2010).

### 2.5 Statistical analysis

The statistical analysis was performed with SPSS 23.0 (IBM, NY, USA). The prevalence (frequencies and percentages) of diabetes (outcome) in Chinese adults was analyzed separately for the two different years (2003 and 2009) and by exposure variables (Table 1). Exposure variables that were significant with chi-square tests were then included in the multivariate regression models (Table 2). Multivariable logistic regression analyses were used to assess the association between exposure variables and diabetes (outcome). The 2003 model (SAGE Wave 0) was adjusted for age and tobacco. The 2009 model (SAGE Wave 1) was adjusted for age, BMI, education and alcohol. All variables were included in the models as categorical variables. Results from the logistic regression analyses are presented as odds ratios (ORs) with 95% confidence intervals (CIs).

**Table 1:** Prevalence of diabetes (outcome) in Chinese adults, by year and exposure variables.

Variables	Categories	2003	2009	Dif. % (2009–2003)
Overall	–	67 (1.7)	770 (8.1)	6.4
Sex	Females	40 (2.0)	413 (8.1)	6.1
	Males	27 (1.4)	357 (8.1)	6.7
Age 2003, 2009	< 60 years	33 (1.0)	241 (5.1)	4.1
	≥ 60 years	34 (4.4)	529 (11.0)	6.6
BMI 2009	<25.0: Underweight / Normal weight	54 (1.6)	370 (6.8)	5.2
	25.0–29.9: Overweight	13 (2.5)	277 (9.8)	7.3
	≥30.0: Obesity	0 (0.0)	87 (14.7)	14.7
Marital status	Never married, Separated/Divorced, Widowed	14 (1.9)	121 (8.2)	6.3
	Currently married, Co-habiting	53 (1.6)	649 (8.1)	6.5
Education 2009	Less than primary school, Primary school completed	31 (1.8)	274 (9.1)	7.3
	Secondary school completed, High school (or equivalent) completed	29 (1.6)	325 (7.3)	5.7
	College / pre-university / university completed, Post graduate degree completed	7 (1.5)	72 (9.6)	8.1
Tobacco 2003	Daily	7 (0.7)	137 (7.3)	6.6
	Yes, but not daily	5 (1.9)	14 (7.7)	5.8
	No, not at all	55 (2.0)	39 (9.6)	7.6
Alcohol 2009	Yes	12 (1.1)	117 (6.5)	5.4
	Never	55 (1.9)	641 (8.4)	6.5
Fruit & vegetables	< 5 servings/day	65 (1.8)	114 (7.8)	6.0
	≥ 5 servings/day	2 (0.7)	619 (8.2)	7.5
Physical activity	< 600 MET-minutes/week	3 (1.0)	103 (7.6)	6.6
	≥ 600 MET-minutes/week	60 (1.7)	357 (9.1)	7.4

Values expressed in Frequencies (Valid %). “Valid percent” is the percent when missing data are excluded from the calculations.

Significant differences between groups were calculated with chi-square tests.

The exposure variables that were significant in predicting diabetes (outcome) were included in the regression models (Table 2).

2003 Significant differences in the prevalence of diabetes in 2003.

2009 Significant differences in the prevalence of diabetes in 2009.

**Table 2:** Associations between exposure variables and diabetes (outcome) in Chinese adults, estimated by multivariable logistic regression (by year).

Variables	Categories	2003	2009
Sex	REF: Females	**–**	**–**
	Males	**–**	**–**
Age ^2003, 2009^	REF: < 60 years	1.0	1.0
	≥ 60 years	4.344 (2.670–7.070) ***	2.454 (2.060–2.924) ***
BMI ^2009^	REF: <25.0: Underweight / Normal weight	–	1.0
	25.0–29.9: Overweight	–	1.422 (1.193–1.694) **
	≥30.0: Obesity	–	2.118 (1.609–2.787) ***
Marital status	Never married, Separated/Divorced, Widowed	–	–
	REF: Currently married, Cohabiting	–	–
Education ^2009^	Less than primary school, Primary school completed	–	0.953 (0.716–1.268)
	Secondary school completed, High school (or equivalent) completed	–	0.931 (0.703–1.233)
	REF: College / pre-university / university completed, Post graduate degree completed	–	1.0
Tobacco ^2003^	Daily	0.375 (0.170–0.827) *	–
	Yes, but not daily	1.045 (0.412–2.652)	–
	REF: No, not at all	1.0	–
Alcohol ^2009^	Yes	–	0.722 (0.579–0.901) **
	REF: Never	–	1.0
Fruit & vegetables	< 5 servings/day	–	–
	REF: ≥ 5 servings/day	–	–
Physical activity	< 600 MET-minutes/week	–	–
	REF: ≥ 600 MET-minutes/week	–	–

Values expressed in Odds Ratio (95% Confidence Interval). * P < 0.05. ** P < 0.01. *** P < 0.001. REF: reference category.

2003: Logistic regression analyses were adjusted for age and tobacco.

2009: Logistic regression analyses were adjusted for age, BMI, education and alcohol.

For the 2003 data, the missing data were: physical activity (n = 4; 6.0%). The missing data in the people with diabetes in 2009 were: BMI (n = 36; 4.7%), education (n = 99; 12.9%), tobacco (n = 580; 75.3%), alcohol (n = 12; 1.6%), fruit and vegetables (n = 37; 4.8%) and physical activity (n = 310; 40.3%). Complete-case analysis was carried out. The data from 2009 had a high percentage of missing data in the variables tobacco (75.3%) and physical activity (40.3%). However, these would not affect the multivariate regression analyses as they were not significant in the bivariate analyses and therefore, they were not included in the regression model. The level of statistical significance was set at p < 0.05.

## 3 Results

The overall prevalence of diabetes in Chinese adults changed from 1.7% in 2003 (total sample: 3993; sample with diabetes: 67) to 8.1% in 2009 (total sample: 9524; sample with diabetes: 770), showing a significant increase of 6.4% within just 6 years. Therefore, the temporal change in prevalence of diabetes was 4.76 times higher in 2009 in comparison to 2003.

In the bivariate analyses (Table 1), the exposure characteristics that were significantly (p < 0.05) associated with diabetes in 2003 were older age ≥ 60 years (4.4%) and no tobacco consumption (2.0%). In 2009, the exposure characteristics significantly associated with diabetes were older age ≥ 60 years (11.0%), obesity (14.7%), higher education (9.6%) and no alcohol consumption (8.4%). Adjusted multivariable logistic regression analyses (Table 2) retained older age ≥ 60 years (OR 4.344, 95% CI 2.670–7.070) in 2003. Interestingly, daily tobacco consumption was associated with a lower risk of diabetes (OR 0.375, 95% CI 0.170–0.827). In 2009, the exposure characteristics significantly associated with diabetes were older age ≥ 60 years (OR 2.454, 95% CI 2.060–2.924), being overweight (OR 1.422, 95% CI 1.193–1.694) and obese (OR 2.118, 95% CI 1.609–2.787). In addition, alcohol consumption was associated with a lower risk of diabetes (OR 0.722, 95% CI 0.579–0.901). Importantly, the odds ratio for older age groups decreased by nearly half (4.344 vs. 2.454) in 2009 compared to 2003, with obesity and being overweight retained as significant risk factors.

## 4 Discussion

This is the first study analysing the temporal changes in prevalence of diabetes and associated risk and protective factors in China between 2003 and 2009. Unlike prevalence studies, this study shows the change in prevalence of diabetes at different time points and significant associations with each timeframe were assessed. This adds an evolutionary perspective of risk factors associated with the change in prevalence of diabetes in China.

A significant increase of 4.76 X prevalence of diabetes is shown between 2003 (1.7%) and 2009 (8.1%). Recent research suggests that this has increased even further, with latest figures of 9.2 % in 2019 from the International Diabetes Federation (2019). While older age was shown to be a significant risk factor both in 2003 and 2009, it is worth noting that the Odds Ratio associating diabetes and older age is nearly half in 2009 data compared to 2003 data (ORs 2.454 vs. 4.344 respectively). On the other hand, obesity and increased weight were significantly associated with the higher prevalence, suggesting a possible significant association with changes in life style.

The association between older age and diabetes is known (Laakso and Pyorala, 1985, Yang et al., 2016, American Diabetes Association, 2020). This may be explained directly by the aging process itself affecting the older pancreas and reduced insulin production, or indirectly through several other age-related risk factors of diabetes such as mitochondrial dysfunction, free fatty acids and lipid metabolisms disorders, inflammation, β-cell dysfunction, insulin resistance, metabolic syndrome, or other factors (Suastika et al., 2012). The associated risk between obesity and diabetes is also well known (Leong and Wilding, 1999, Scheen, 2000). Plasma leptin, tumour necrosis factor-α and non-esterified fatty acid levels are all elevated in obesity and play a role in insulin resistance and diabetes (Leong and Wilding, 1999). Specifically, in China the demographics of the ageing population and obesity, possibly due to changes in life style, are increasing fast (Mai et al., 2013, Zeng et al., 2021), being two important factors that have been linked to an increase the prevalence of diabetes globally.

Our results show a negative association between tobacco consumption and diabetes. Whilst it widely known that tobacco increases the risk of diabetes (Pan et al., 2015, White et al., 2018, Akter et al., 2015), some studies also report a negative association as shown by our data (Hou et al., 2016, Liu et al., 2017). It is likely that people who have diabetes have been advised to stop smoking by a doctor. Literature suggests other reasons to explain the negative association of tobacco use and diabetes. Smoking is associated to a decreased weight (Audrain-McGovern and Benowitz, 2011) through increased energy expenditure (Hofstetter et al., 1986, Collins et al., 1996) and suppresses appetite (Seeley and Sandoval, 2011), that may be associated with decreased weight and a reduced risk of diabetes (Leong and Wilding, 1999, Scheen, 2000). In addition, it has been suggested that cigarette smoking may reduce stress (Parrott, 1999, Choi et al., 2015) which is an important risk factor for diabetes and chronic hyperglycemia (Surwit et al., 1992). It is really important that the significant negative association with diabetes as shown by our study and other studies is treated with caution and more work needs to be done to examine this fully.

The results of this study also showed alcohol consumption to be negatively associated with the diabetes. Other studies have also shown this and literature suggests that light-to-moderate alcohol consumption decreases the incidence of diabetes, while heavy and binge drinking show an increased risk (Polsky and Akturk, 2017). The mechanisms for this are not completely clear yet (Pietraszek et al., 2010) and more work needs to be done to fully understand how alcohol consumption may affect insulin sensitivity (Facchini et al., 1994, Mayer et al., 1993) or the modulation of changes in the endocrine functioning of fat tissue, the inflammatory status of several organs and/or the modulation of glucose and fatty acid metabolism (Hendriks, 2007). It is also possible that those who have diabetes have been told to stop drinking alcohol by a doctor and this may explain the results obtained. The WHO survey does not indicate the level of alcohol consumed and this needs further investigation.

The main strengths of this study are the nationally representative WHO sample of Chinese adults with diabetes with the same questionnaire, so temporal changes over the two years can be adequately compared, the nearly equal gender distribution and the wide age range covered. However, as with all cross-sectional surveys, some limitations should be considered. First, the cross-sectional design does not allow to establish causality. Second, diabetes was self-reported in the survey, and data on the type of diabetes (i.e., type 1 or type 2) was not available. Third, BMI was self-reported too; although wave 1 had measured weight and height too, there were many missing data in that variable and the self-reported option was preferred to avoid losing many data and to have the same measurement methods in the two waves; it should be noted that BMI computed from self-reported weight and height is a valid measure (Hodge et al., 2020). Fourth, SAGE only included individuals with a valid home address, and this may have impacted the study results. Finally, although the questionnaire IPAQ to assess physical activity, used in SAGE Wave 0 (2003), is designed for adults up to 65 years, the survey used this instrument on people older than 65 years, however literature suggests that IPAQ is an adequate and useful tool for assessing physical activity among elderly adults (Tomioka et al., 2011). We recommend that future studies continue to analyse the changing profile of diabetes in China and the risk factors that influence it. If possible, longitudinal studies should be conducted with the same population and measure as many variables as possible objectively instead of using self-report.

## 5 Conclusion

According to the WHO SAGE data in 2003 and 2009, the prevalence of self-reported diabetes in Chinese adults increased by 4.76 times from 2003 to 2009. In 2003, older age ≥ 60 years was the only significant risk factor, while in 2009 the odds ratio associated with older age ≥ 60 years decreased and obesity and excess weight were retained as significant risk factors. These findings have important implications for public health in China. Public health strategies should take into account the significant life style risk factors linked to obesity and weight gain, which are associated with the higher prevalence of diabetes. Public health strategies, such as educational and psychological interventions, should pay special attention to older adults and people with excess weight or obesity.

## CRediT authorship contribution statement

**Shahina Pardhan:** Writing – original draft. **Dingchan Zheng:** Writing – review & editing. **Zhiqing Chen:** Writing – review & editing. **Guillermo F. López Sánchez:** Writing – original draft.

## Declaration of Competing Interest

The authors declare that they have no known competing financial interests or personal relationships that could have appeared to influence the work reported in this paper.
